# Effect of Recombinant Human Amelogenin on the Osteogenic Differentiation Potential of SHED

**DOI:** 10.3390/cells14090657

**Published:** 2025-04-30

**Authors:** Akira Hirabae, Ryo Kunimatsu, Yuki Yoshimi, Kodai Rikitake, Shintaro Ogashira, Ayaka Nakatani, Shuzo Sakata, Kotaro Tanimoto

**Affiliations:** Department of Orthodontics, Applied Life Sciences, Institute of Biomedical & Health Sciences, Hiroshima University, Hiroshima 734-8553, Japan; d224959@hiroshima-u.ac.jp (A.H.); yukimihsoy@hiroshima-u.ac.jp (Y.Y.); shuna-s0102@hiroshima-u.ac.jp (K.R.); milk595@hiroshima-u.ac.jp (S.O.); anakatan@hiroshima-u.ac.jp (A.N.); shuzosakata@hiroshima-u.ac.jp (S.S.); tkotaro@hiroshima-u.ac.jp (K.T.)

**Keywords:** amelogenin, rh174, SHED, MSCs, osteoblast differentiation, calcification

## Abstract

This study aimed to explore how amelogenin can improve stem cells from human exfoliated deciduous teeth (SHED)–based bone regeneration and promote tissue healing as a treatment for critical-sized bone defects. SHED was induced into bone differentiation by using osteogenic differentiation medium. Real-time polymerase chain reaction, alkaline phosphatase (ALP) staining and quantification, and Alizarin Red S staining, as well as calcium and osteocalcin quantification were performed to assess differentiation. On day 18, a significant increase was observed in the expression of *RUNX2*, *CBFB*, *BGLAP*, *COL1*, *BMP2*, *BMP4*, *NOTCH1*, *NOTCH2*, and *NES*. Osteocalcin gene expression continued to increase significantly. ALP activity was significantly higher in the amelogenin-treated group than in the control group on days 7, 10, and 14. On day 14, enhanced ALP staining was observed in the amelogenin-treated group. Calcium and osteocalcin levels were significantly higher in the amelogenin-treated group than in the control group on day 21. This study suggests that combining SHED and amelogenin may be effective for bone regeneration, offering a potential new approach in regenerative medicine.

## 1. Introduction

Critical-sized bone defects, commonly encountered in dentistry, are bone defects that do not heal spontaneously. These include defects caused by periodontal disease, jaw-area resection due to tumors, and cleft palate (CLP) conditions [1]. For patients with CLP conditions, secondary alveolar bone grafts using fresh autogenous bone grafting just before canine tooth eruption has become the gold standard for CLP closure because it leads to favorable outcomes. This approach has been adopted by approximately 90% of centers in North America [2]. The iliac bone is the most favored donor site for fresh autogenous bone grafts [3]. However, iliac bone grafting for CLP is invasive and poses significant risks to younger patients. These risks include gait disturbance due to postoperative pain, hematoma, iliac crest deformity, infection, pelvic fracture, lateral femoral cutaneous nerve injury, and resorption of the grafted bone [4,5,6]. Therefore, alternative therapies should be established.

The concept of the Tissue Engineering Triad has been recently proposed to improve the efficiency of tissue regeneration and consists of three components: a cell source useful for tissue healing and differentiation; growth factors that act on cells and make tissue healing more efficient [7]; and biomaterials that harmonize with the surrounding tissue, assist tissue healing, provide mechanical strength, and have biological activity [8].

Mesenchymal stem cells (MSCs) have been isolated from various tissues since their isolation from bone marrow was reported in 1970 [9]. MSCs are expected to be used in regenerative medicine in various tissues owing to their high proliferative potential and their ability to differentiate into different cells, including chondrocytes, adipocytes, and bone cells. MSCs from the bone marrow, adipose tissue, dental pulp, and umbilical cord have been suggested to be useful in bone tissue engineering [10] and are considered a promising cell source in bone tissue engineering because of their potential for osteogenic differentiation, immunomodulatory, and cytokine secretion functions [11]. Recently, stem cells from human exfoliated deciduous teeth (SHED) have attracted increasing interest in dentistry owing to their potential in regenerative medicine. SHED were first isolated by Miura, Gronthos, Zhao, Lu, Fisher, Robey, and Shi [12] and have since attracted attention because of their origin from deciduous teeth, which are naturally replaced by permanent teeth. Harvesting deciduous dental pulp to obtain SHED is less invasive than other methods of obtaining MSCs, such as bone marrow stem cells through marrow puncture. CLP bone grafting is typically performed at around 9 years of age to facilitate canine tooth eruption. At this stage, patients often retain deciduous teeth, which can be harvested for SHED. SHED can be collected from severely carious deciduous teeth [13] with a high success rate of isolation [14], making SHED a promising cell source for tissue regeneration because of the ease and feasibility of collection.

Stimulating MSC differentiation with growth factors is more effective for tissue regeneration than transplanting MSCs alone without any modification. One such growth factor is the enamel matrix derivative (EMD), specifically Emdogain^®^, which has been investigated in dentistry [15,16]. EMD promotes wound healing in human periodontal tissues [17]. However, given that Emdogain^®^ is a preparation of unknown composition derived from young porcine tooth embryos, it raises concerns because of potential risks, such as the presence of unknown pathogens and various proteins of unknown function that may not contribute to healing promotion.

Amelogenin, the main component of Emdogain^®^, has been suggested to be a useful auxiliary factor with hard-tissue induction properties [18,19,20,21]. Amelogenin accounts for approximately 90% of the enamel matrix proteins during the early stages of tooth development [22], suggesting its crucial role in tooth calcification. In the medical field, Xelma^®^ has been commercialized as an amelogenin-containing formulation [23]. Although amelogenin exhibits physiological activity in MSCs and has been clinically applied in the medical field, there are no documented examples of its clinical use as a biologically active substance in dentistry. Furthermore, the specific effects of amelogenin on SHED have yet to be clarified.

This study aimed to investigate the influence of amelogenin on the efficiency of SHED-based tissue regeneration in bone tissue engineering, with a focus on the bone regenerative capacity.

## 2. Materials and Methods

### 2.1. Cell Culture

Deciduous teeth were extracted from 4 patients (mean age of 12 years 6 months, ±1 year 10 months), whose informed consent was obtained in strict compliance with the regulations for epidemiological research at Hiroshima University Hospital (E2015-0020-02) [12]. SHED were isolated and cultured according to the method of Gronthos et al. [24], and the SHED used in this study were obtained from the maxillary deciduous canines, maxillary second deciduous molars, and mandibular second deciduous molars. The extracted deciduous teeth were immersed in phosphate-buffered saline (PBS) containing 100 mM amphotericin and immediately placed in a culture room. After disinfection with isodine (Maruishi Pharmaceuticals, Osaka, Japan) and Hibiten (Dainippon Sumitomo Pharma, Osaka, Japan), the periapical tissue around the extracted tooth was removed using a scalpel. After re-disinfection, the deciduous tooth was divided using osteoclastic forceps (Natsume Corporation, Tokyo, Japan) with the cemento–enamel junction as a guide, and the pulp tissue was collected. The pulp tissue was then immersed in a solution of 4 mg/mL collagenase (Thermo Fisher Scientific, Waltham, MA, USA) and 3 mg/mL dispase (Godo Shusei, Tokyo, Japan) in minimal essential medium Eagle (α-MEM; Sigma-Aldrich, St. Louis, MO, USA) and physically shredded with a scalpel. Incubation was then carried out at 37 °C for 50 min under 5% CO_2_ with shaking. After enzyme treatment, cell aggregates were removed in a 70 µm cell strainer, and the filtered solution was diluted with α-MEM and centrifuged at 1500 rpm for 5 min. The supernatant was aspirated and incubated with 20% fetal bovine serum (FBS; SERANA, Brandenburg, Germany), 0.24 µL/mL kanamycin (Meiji Seika Pharma, Tokyo, Japan), 0.5 µL/mL penicillin (Meiji Seika Pharma, Tokyo, Japan), and 1 µL/mL amphotericin, all of which were suspended in α-MEM. The resulting cell suspension was then seeded into 35 mm cell culture Petri dishes (CORNING, Corning, NY, USA). The cells were incubated under 37 °C in a 5% CO_2_ atmosphere. Once confluence was confirmed, cells were passaged using PBS containing 0.25% trypsin (Nacalai tesque Corporation, Kyoto, Japan) and 1 mM EDTA (Wako Pure Chemical Industries, Osaka, Japan). Cultures were incubated with 10% FBS, 2.5 mL penicillin–streptomycin mixture (Nacalai tesque Corporation, Kyoto, Japan; final concentration of 50 U/mL for penicillin and 50 µg/mL for streptomycin, and 1 µL/mL amphotericin in α-MEM at 37 °C under 5% CO_2_ conditions. Cells from passages 4–6 were used for the experiments. After cell culturing, cells were seeded onto 6-well plates (CORNING, Corning, NY, USA) coated with type I collagen derived from porcine tendon for plate-coating (Functional Peptide Institute, Yamagata, Japan) or 12-well Collagen I-Coated Microplates (AGC Techno Glass, Shizuoka, Japan) at a seeding density of 1.0 × 10^4^ cells/cm^2^ and cultured under the same conditions. The bone differentiation induction medium was prepared by adding 100 nM dexamethasone (Sigma-Aldrich, St. Louis, MO, USA), 0.2 mM ascorbate-phosphate (Sigma-Aldrich, St. Louis, MO, USA), and 10 mM β-glycerolphosphate (Sigma-Aldrich, St. Louis, MO, USA) in Dulbecco’s Modified Eagle’s Medium (Sigma-Aldrich, St. Louis, MO, USA). After reaching 80% confluence, bone differentiation induction medium was changed every 2 days and the culture continued at 37 °C under 5% CO_2_ conditions. Recombinant human full-length amelogenin (SAE0117; Sigma-Aldrich, St. Louis, MO, USA) was used in the experiments. The groups with amelogenin (addition of 1000 ng/mL amelogenin simultaneously with the changing of the bone differentiation induction medium) were designated as the amelogenin-treated groups, whereas the groups without amelogenin addition served as the control groups.

According to the criteria established by the International Society for Cellular Therapy, human MSCs must meet three key conditions: (A) adherence to plastic containers under standard culture conditions; (B) expression of surface markers CD105, CD90, and CD73, while lacking expression of CD14 or CD11b, CD79α or CD19, CD45, CD34, and HLA-DR; and (C) capacity for trilineage differentiation into osteoblasts, cartilage cells, and fat cells. In the present study, the cells isolated from dental pulp adhered to the plastic culture dishes, fulfilling the first criterion. Furthermore, in our previous study conducted using the same isolation protocol [25,26], cells isolated from the pulp were negative for CD271 and CD34 and positive for CD146, CD105, CD73, CD44, CD29, and STRO-1. Flow cytometric analysis performed as a preliminary verification in this study revealed a similar immunophenotype, with positive staining for CD29, CD90, CD73, CD44, and CD146, and negative expression for CD34. Additionally, our prior work also confirmed the trilineage differentiation potential of these cells to differentiate into osteoblasts, cartilage cells, and fat cells, satisfying the third criterion. The current study employed the same isolation and culture procedures, which also aligned with the methods described by Nakajima et al. [25] and Kunimatsu R et al. [26]; therefore, we defined the isolated cells as SHED.

### 2.2. Quantification of Live Cell Number Using Imaging Analysis System

SHED from passages 4 to 6 were seeded in 96-well plates (TPP Techno Plastic Products AG, Trasadingen, Switzerland) at a concentration of 5.0 × 10^3^ cells/well. Cell proliferation was assessed under standard culture conditions (10% FBS α-MEM; 37 °C; 5% CO_2_ environment). Following initial cell adhesion, a stepwise serum reduction protocol was used to synchronize the cell cycle: the culture medium was sequentially replaced with 1% FBS α-MEM, then with 0.1% FBS α-MEM after 10 h, and finally, with serum-free α-MEM after an additional 1.5 h. Thirty minutes after the final medium change, the plates were placed in an Incucyte^®^ S3 Live-Cell Analysis System (Sartorius AG, Göttingen, Germany) which continuously captured images every 2 h over a 72 h period. Cell proliferation data were automatically quantified by the Incucyte^®^ S3 Live-Cell Analysis System (Sartorius AG) and proliferation curves were generated accordingly.

### 2.3. BrdU Assay

SHED (passages 4 to 6) were seeded at a concentration of 5.0 × 10^3^ cells per well in 96-well plates and incubated in 10% FBS α-MEM at 37 °C in a 5% CO_2_ environment until 60% confluence was reached. A stepwise serum reduction protocol for cell cycle synchronization, as described in Section 2.2, was employed. Thirty minutes after the final medium change, cells were exposed to the BrdU labelling reagent provided in a colorimetric cell proliferation ELISA kit (Roche Diagnostics, Basel, Switzerland) and incubated for 48 h at 37 °C in a 5% CO_2_ environment. After incubation, the BrdU reaction substrate solution was added, and absorbance was measured at 375 nm using a Multiskan^®^ FC microplate reader (Thermo Fisher Scientific, Waltham, MA, USA).

### 2.4. Real-Time Polymerase Chain Reaction

SHED were seeded on 6-well plates at a concentration of 9.6 × 10^4^ cells/well and cultured in α-MEM containing 10% FBS until 80% confluence was reached. The culture was then transferred to bone differentiation induction medium and divided into two groups: amelogenin-treated group and control group. After culturing in bone differentiation induction medium, cells were collected on days 7, 10, 14, 18, and 21. The RNeasy^®^ Mini Kit (Qiagen, Valencia, CA, USA) was employed for RNA extraction of the samples. The total RNA was then quantified using a NanoDrop One/Onec spectrophotometer (Thermo Fisher Scientific, Waltham, MA, USA) and its purity was assessed based on the OD260/OD280 ratio, with acceptable purity determined by an A260/A280 absorbance ratio between 1.5 and 2.0. The extracted RNA was then unified (500 ng) and reverse transcribed into cDNA using the ReverTra Ace first-strand cDNA synthesis kit (Toyobo, Osaka, Japan). Subsequently, RT-PCR was performed using Thunderbird SYBR qPCR mix (Toyobo, Osaka, Japan) and the specific primers. Gene expression analysis was performed using the Thermal Cycler Dice Real^®^ Time System for the following genes: runt-related transcription factor 2 (*RUNX2*), core-binding factor subunit beta (*CBFB*), type I collagen (*COL1*), osteocalcin (*BGLAP*), bone morphogenetic protein 2 (*BMP2*), bone morphogenetic protein 4 (*BMP4*), notch receptor 1 (*NOTCH1*), notch receptor 2 (*NOTCH2*), and Nestin (*NES*). The primer sequences used in this study are listed in Table 1.

### 2.5. Alkaline Phosphatase Staining

SHED were seeded into 12-well plates at a concentration of 3.8 × 10^4^ cells/well and cultured in 10% FBS α-MEM until they reached 80% confluent. Subsequently, the cells were cultured in bone differentiation induction medium. On day 14 after the initiation of culture in a bone differentiation induction medium, cells were treated with 4% paraformaldehyde (Nacalai Tesque Corporation, Kyoto, Japan) for 10 min on ice, followed by a 1 min treatment with 50% EtOH/50% acetone (Sigma-Aldrich, St. Louis, MO, USA) for permeabilization. After the removal of the permeate, alkaline phosphatase (ALP) staining solution (Fujifilm Wako Pure Chemicals, Osaka, Japan) was added and incubated for 30 min at 37 °C in a 5% CO_2_ environment.

### 2.6. Western Blot Analysis

SHED were seeded into 6-well plates at a concentration of 9.6 × 10^4^ cells/well and cultured in 10% FBS α-MEM until 80% confluence was reached. Cell cultures were maintained in a bone differentiation induction medium. On day 14 after the start of culture growth in the bone differentiation induction medium, total protein was extracted using RIPA lysis buffer (Nacalai Tesque, Kyoto, Japan) supplemented with protease and phosphatase inhibitors. Cell lysates were centrifuged at 15,000× *g* for 20 min at 4 °C, and the protein concentration was determined using the BCA protein assay kit (Thermo Fisher Scientific, Waltham, MA, USA). Equal amounts of protein were loaded onto precast polyacrylamide gels (e-PAGEL, ATTO, Tokyo, Japan) and electrophoresed. They were then transferred to polyvinylidene fluoride membranes by using an iBlot^®^ 2 dry blotting system (Thermo Fisher Scientific, Waltham, MA, USA) according to the manufacturer’s instructions. The membrane was then blocked with 5% skimmed milk (Wako Pure Chemical Industries, Osaka, Japan) for 30 min at room temperature, followed by incubation with primary antibodies overnight at 4 °C. Primary antibodies were anti-ALP (Sc-28904; Santa Cruz Biotechnology, Santa Cruz, CA, USA) and anti-β-actin (Wako Pure Chemical Industries, Osaka, Japan). The secondary antibodies, either Alexa Fluor^TM^ 488 donkey anti-mouse IgG (Thermo Fisher Scientific, Waltham, MA, USA) or Alexa Fluor^TM^ 594 goat anti-rabbit secondary antibody (Thermo Fisher Scientific, Waltham, MA, USA), were incubated at room temperature for 1 h. Protein bands were visualized using Licor Odyssey CLx^®^ (LI-COR Biosciences, Lincoln, NE, USA). The obtained bands were quantified in ImageJ software (NIH, Washington, DC, USA).

### 2.7. ALP Quantification

SHED were seeded into 6-well plates at a concentration of 9.6 × 10^4^ cells/well and cultured in 10% FBS α-MEM until 80% confluence was reached. Cell cultures were maintained in a bone differentiation induction medium. On days 7, 10, and 14 after the start of culture in the bone differentiation induction medium, cells were collected and sonicated on ice using a Sonic Vibra Cell (Sonic & Materials, Newtown, CT, USA). The samples were then centrifuged at 2500× *g* for 10 min, and the supernatant was collected. ALP was quantified using the pNPP alkaline phosphatase assay (AnaSpec, Fremont, CA, USA). A 50 µL sample was added to a 96-well plate, followed by the addition of 50 µL of pNPP substrate solution. The plate was then shaken and incubated for 1 h before analysis. The absorbance was then measured at a wavelength of 405 nm using a Multiskan^®^ FC microplate reader (Thermo Fisher Scientific, Waltham, MA, USA). Quantitative data for ALP were quantified by creating a calibration curve using the ALP Standard supplied with the ELASA kit and applying the absorbance of the measured samples.

### 2.8. Alizarin Red S Staining

SHED were seeded onto a 12-well plate Collagen I-Coated Microplate at a concentration of 3.8 × 10^4^ cells/well and cultured in 10% FBS α-MEM until 80% confluence was reached. The medium was then replaced with bone differentiation induction medium, and the culture was continued. On day 21 of incubation in the bone differentiation induction medium, the medium was aspirated, and each well was washed once with PBS. Cells were then fixed with 4% paraformaldehyde on ice for 10 min, and each well was washed three times with purified water. After staining each well using the Calcification Evaluation Kit (PG Research Inc., Tokyo, Japan), 500 µL/well of the calcification nodule lysate in the kit was added, shaken, and stirred for 10 min. Thereafter, 100 µL of the resulting eluate was transferred to a 96-well plate, and the absorbance was measured at a wavelength of 405 nm using a microplate reader.

### 2.9. Calcium Quantification

SHED were seeded into 6-well plates at 9.6 × 10^4^ cells/well and cultured in 10% FBS α-MEM until 80% confluence was reached. Culture medium was then replaced with bone differentiation induction medium and maintained for 21 days. After incubation, each well was washed once with 10 mM Tris-HCl buffer (pH 7.0) (Nacalai Tesque Corporation, Kyoto, Japan). Then, 500 µL of 10% formic acid (Nacalai Tesque Corporation, Kyoto, Japan) solution was added to each well, shaken, and stirred for 8 h at 4 °C. The solution was then collected as a sample. Analysis was performed using the Calcium E Test (Wako Pure Chemical Industries, Osaka, Japan). A 5 µL sample was added to a 96-well plate and 190 µL of a 2:1 mixture of buffer and chromogenic solution from the kit was added. Absorbance was then measured at a wavelength of 620 nm using a microplate reader.

### 2.10. Osteocalcin Quantification

SHED were seeded into 6-well plates at a concentration of 9.6 × 10^4^ cells/well and cultured in 10% FBS α-MEM until 80% confluence was reached. Cell cultures were maintained in a bone differentiation induction medium. On day 21 after the start of culture in bone differentiation induction medium, the cell culture supernatant was collected, centrifuged at 2000× *g* for 10 min, and separated from the solid material. Only the supernatant was collected and used as a sample. Osteocalcin was quantified using the Human Osteocalcin SimpleStep ELISA^®^ Kit (ab270202; Abcam, Cambridge, MA, USA). The absorbance of the color products was quantitatively measured using a microplate reader Multiskan^®^ FC at a wavelength of 450 nm. A calibration curve was used for quantification. OCN quantitative data were quantified by creating a calibration curve using the OCN Standard supplied with the ELASA kit and applying the absorbance of the measured samples.

### 2.11. Statistical Analysis

Means and standard deviations were calculated and presented to quantify the results. Comparisons between the two groups were performed using Welch’s *t*-test, and Tukey’s HSD test was used for multiple comparison. Statistical significance was set at *p* < 0.05.

## 3. Results

### 3.1. Cell Morphology

Individual cells were spindle-shaped, and the plated culture formed a herringbone pattern.

### 3.2. Quantification of Live Cell Number

**Figure 1:**
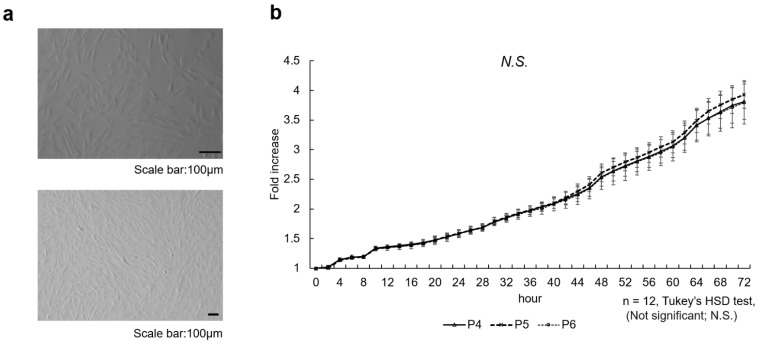

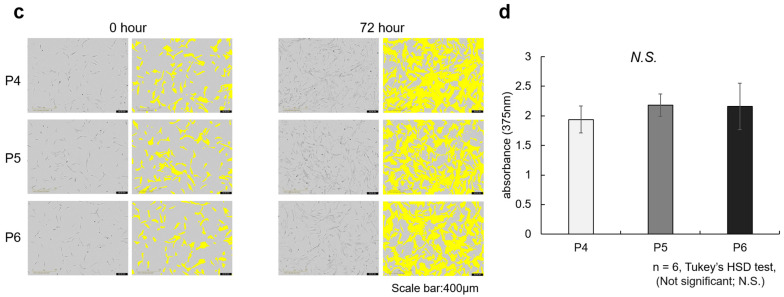
Characteristics of the SHED used in the experiments.

The cell proliferation of SHED from passages 4 to 6 did not differ significantly at any time point from one another (Figure 1b) (n = 12, Tukey’s HSD test, not significant [NS]). Figure 1c shows representative images obtained at the start of the recording and 72 h later. The yellow areas are those recognized as cells by the Incucyte^®^ S3 Live-Cell Analysis System.

### 3.3. BrdU Assay

The BrdU assay showed no significant differences between SHED passages 4 to 6 (Figure 1d) (n = 6, Tukey’s HSD test, NS).

(a) Representative light microscopy images of stem cells from human exfoliated deciduous teeth (SHED). SHED were isolated and cultured from deciduous teeth of patients according to the method of Gronthos et al. Cell cultures were grown in α-MEM containing 10% FBS, 50 U/mL penicillin, 50 µg/mL streptomycin, and 1 µL/mL amphotericin under 37 °C and 5% CO_2_ conditions. Individual cells were spindle-shaped, and the plated culture formed a herringbone pattern. Cell morphology did not change with successive passages. Scale bar 100 µm.
(b) Cell proliferation curves obtained by Incucyte^®^ S3 Live-Cell Analysis System. SHED from passages 4 to 6 were seeded in 96-well plates at 5.0 × 10^3^ cells/well and cell proliferative capacity was compared in a 10% FBSα-MEM environment. Cells were photographed and assessed every 2 h for a total of 72 h using an Incucyte^®^ S3 Live-Cell Analysis System. No significant differences were found between passage 4, 5, and 6 cells at any time point (n = 12, Tukey’s HSD test, not significant [NS]).
(c) Representative images obtained at the start of the recording and 72 h later. The yellow areas were recognized as cells by the Incucyte^®^ S3 Live-Cell Analysis System. Scale bar 400 µm.
(d) Quantification of passages 4 to 6 SHED using colorimetric absorbance of 5-bromo-2-deoxyuridine uptake. SHED from passages 4 to 6 were seeded at 5.0 × 10^3^ cells/well and incubated in 10% FBS α-MEM at 37 °C in a 5% CO_2_ environment until 60% confluence was reached. The cells were assessed using a cell proliferation ELISA and BrdU colorimetric kit. No significant differences were observed between SHED passages 4 to 6 (n = 6, Tukey’s HSD test, NS).

### 3.4. Evaluation of Gene Expression of Human Full-Length Amelogenin in SHED During Induction of Bone Differentiation

**Figure 2:**
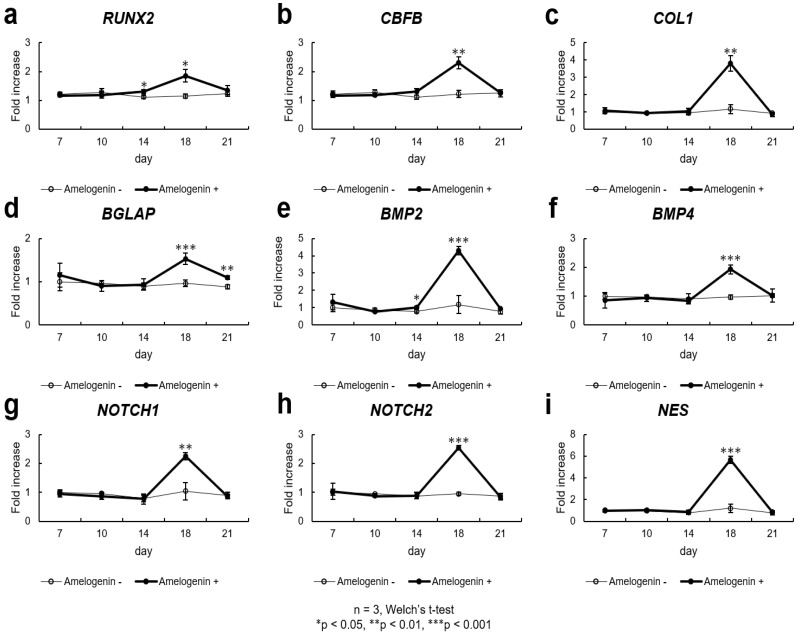
The effect of amelogenin addition on the expression of osteogenesis-related markers in SHED.

On day 14 after the induction of bone differentiation, a significant increase in *RUNX2* and *BMP2* gene expression was observed, and on day 18 a significant increase was observed in *RUNX2*, *CBFB*, *BGLAP*, *COL1*, *BMP2*, *BMP4*, *NOTCH1*, *NOTCH2*, and *NES* gene expression. Moreover, a significant increase in *BGLAP* gene expression was observed on day 21 after the induction of bone differentiation (n = 3, Welch’s *t*-test, * *p* < 0.05, ** *p* < 0.01, *** *p* < 0.001), Figure 2.

(a) SHED were seeded at 9.6 × 10^4^ cells/well and cultured in 10% FBS αMEM. Upon reaching 80% confluence, the culture was replaced with osteogenesis induction medium, and amelogenin was added at a concentration of 1000 ng/mL. The expression of osteogenesis-related markers was assessed by quantitative real-time polymerase chain reaction, revealing increased expression of all mRNA levels (n = 3, Welch’s *t*-test, * *p* < 0.05). On day 14 after the induction of bone differentiation, a significant increase in *RUNX2* gene expression was observed. and on day 18 a significant increase was observed in *RUNX2* gene expression (n = 3, Welch’s *t*-test, * *p* < 0.05).
(b) On day 18 a significant increase was observed in *CBFB* gene expression (n = 3, Welch’s *t*-test, ** *p* < 0.01).
(c) The mRNA expression levels of *COL1* in SHED were upregulated significantly (*p* < 0.01) by the treatment with amelogenin on day 18 (n = 3, Welch’s *t*-test, ** *p* < 0.01).
(d) On day 18 a significant increase was observed in *BGLAP* gene expression. Moreover, a significant increase in *BGLAP* gene expression was observed on day 21 after the induction of bone differentiation (n = 3, Welch’s *t*-test, ** *p* < 0.01, *** *p* < 0.001).
(e) On day 14 a significant increase was observed in *BMP2* gene expression. Moreover, on day 18 a significant increase was observed in *BMP2* gene expression (n = 3, Welch’s *t*-test, * *p* < 0.05, *** *p* < 0.001).
(f) A significant increase in *BMP4* gene expression was observed on day 18 after the induction of bone differentiation (n = 3, Welch’s *t*-test, *** *p* < 0.001).
(g) The expression of *NOTCH1* was significantly enhanced by amelogenin in SHED compared with that in the control groups on day 18 (n = 3, Welch’s *t*-test, ** *p* < 0.01).
(h) A significant increase in *NOTCH2* gene expression was observed on day 18 after the induction of bone differentiation (n = 3, Welch’s *t*-test, *** *p* < 0.001).
(i) The mRNA expression levels of *NES* in SHED were upregulated significantly (*p* < 0.001) by the treatment with 1000 ng/mL amelogenin on day 18 (n = 3, Welch’s *t*-test, *** *p* < 0.001).

### 3.5. Evaluation of ALP Staining

On day 14 after the start of osteoblast differentiation, ALP staining was more pronounced in the amelogenin-treated group than in the control group.

### 3.6. Evaluation of ALP Activity

On days 7, 10, and 14 after the start of osteoblast differentiation, ALP activity was significantly higher in the amelogenin-treated group than in the control group (n = 6, Welch’s *t*-test; * *p* < 0.05).

### 3.7. Evaluation of Western Blot Analysis

**Figure 3:**
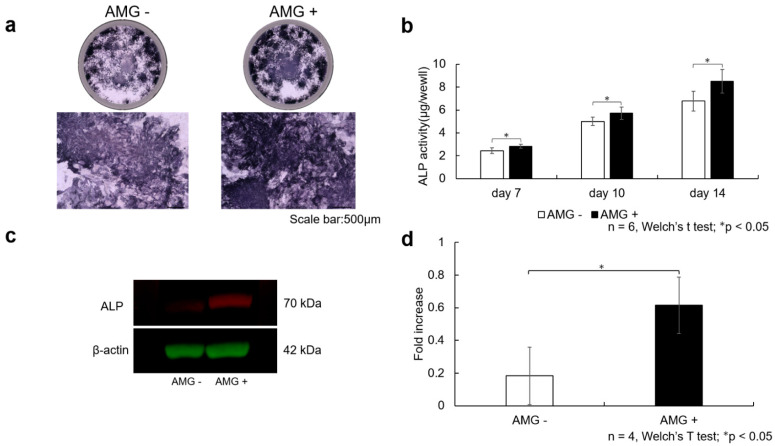
The effect of amelogenin addition on ALP activity in SHED, detail in Supplementary Materials.

On day 14 after the start of osteoblast differentiation, Western blot analysis showed that amelogenin treatment induced ALP protein expression in SHED, Figure 3.

(a) SHED were seeded at 3.8 × 10^4^ cells/well and cultured in 10% FBS αMEM. After reaching 80% confluence, the culture was replaced with osteogenesis induction medium, and amelogenin was added at a concentration of 1000 ng/mL. Scale bar is shown at 500 µm. The amelogenin-treated group demonstrated more enhanced ALP staining than the control group.
(b) ALP activity was also assessed using the pNpp Alkaline Phosphatase Kit, revealing increased ALP activity at 7, 10, and 14 days after the start of osteogenesis. Data are expressed as the absorbance (405 nm). A more significant enhancement in ALP activity was observed in the amelogenin-treated group than in the control group (n = 6, Welch’s *t*-test; * *p* < 0.05).
(c) After SHED were stimulated for 14 days in bone differentiation induction medium supplemented with amelogenin (1000 ng/mL), total protein was collected, and Western blot analysis was performed to confirm ALP protein expression. β-actin was used as the loading control. Increased ALP protein levels were observed in the amelogenin-treated group compared with the control group.
(d) The obtained bands were quantified using ImageJ software (n = 4, Welch’s *t*-test; * *p* < 0.05).

### 3.8. Evaluation of Alizarin Red S Staining

On days 14 and 21 after osteoblast differentiation, staining was more enhanced in the amelogenin-treated group than in the control group. This enhancement also significantly increased when quantified by absorbance measurement (n = 6, Welch’s *t*-test, ** *p* < 0.01, *** *p* < 0.001).

**Figure 4:**
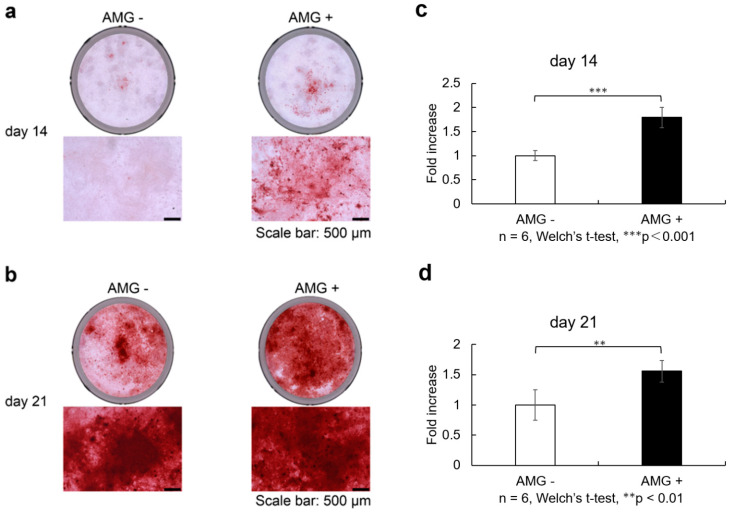
Effect of amelogenin addition on SHED calcification, detail in Supplementary Materials.

SHED were seeded at 9.6 × 10^4^ cells/well and cultured in 10% FBS αMEM. Upon reaching 80% confluence, the culture was replaced with osteogenesis induction medium, and amelogenin was added at a concentration of 1000 ng/mL. Alizarin red S staining was performed at 14 and 21 days of culture in the bone differentiation induction medium. Scale bars are shown at 500 µm. Staining was more enhanced in the amelogenin-treated group than in the control group (Figure 4a,b). Calcification nodule lysate was added, and the absorbance of the resulting colored material was measured and shown as data (405 nm). Calcification was more evident in the amelogenin-treated group than in the control group (Figure 4c,d) (n = 6, Welch’s *t*-test, ** *p* < 0.01, *** *p* < 0.001).

(a) SHED was seeded and cultured in 10% FBS αMEM. Upon reaching 80% confluence, the culture was replaced with osteogenesis induction medium, and amelogenin was added at a concentration of 1000 ng/ml. Alizarin red S staining was performed at 14 and 21 days of culture in the bone differentiation induction medium. Scale bars are shown at 500 µm. On days 14 after osteoblast differentiation, staining was more enhanced in the amelogenin-treated group than in the control group.
(b) On days 21 after osteoblast differentiation, staining was more enhanced in the amelogenin-treated group than in the control group.
(c) Staining was more enhanced in the amelogenin-treated group than in the control group. Calcification nodule lysate was added, and the absorbance of the resulting coloured material was measured. Calcification was more evident in the amelogenin-treated group than in the control group. (n = 6, Welch’s *t*-test, *** *p* < 0.001).
(d) On days 21 after osteoblast differentiation, calcification was more evident in the amelogenin-treated group than in the control group (n = 6, Welch’s *t*-test, ** *p* < 0.01).

### 3.9. Evaluation of Calcium Quantification

On day 21 after osteoblast differentiation, significantly higher calcium levels were observed in the amelogenin-treated group than those in the control group (n = 6, Welch’s *t*-test, ** *p* < 0.01).

### 3.10. Evaluation of Osteocalcin Quantification

**Figure 5:**
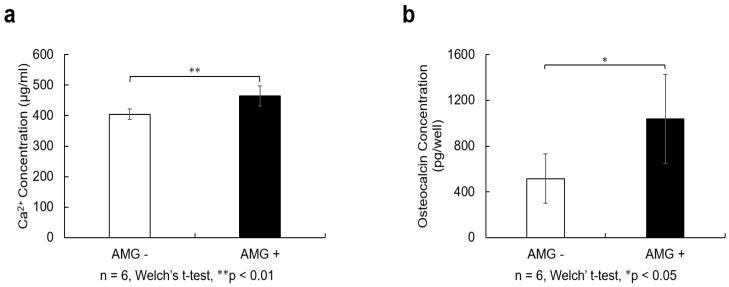
Effect of amelogenin addition on calcium deposition in SHED.

On day 21 after osteoblast differentiation, osteocalcin levels were significantly higher in the amelogenin-treated group than in the control group (n = 6, Welch’s *t*-test, * *p* < 0.05), Figure 5.

(a) SHED were seeded at 9.6 × 10^4^ cells/well and cultured in 10% FBS αMEM. After reaching 80% confluence, the culture was replaced with osteogenesis induction medium, and amelogenin was added at a concentration of 1000 ng/mL. Twenty-one days after the start of osteoblast differentiation induction, 10% formic acid solution was added to cells and shaken at 4 °C for 8 h. The resulting lysate was used for calcium quantification. The amelogenin-treated group showed higher calcium content than the control group (n = 6, Welch’s *t*-test; ** *p* < 0.01).
(b) Cell culture supernatants were collected from the cells 21 days after the induction of osteoblast differentiation and centrifuged at 2000× *g* for 10 min. The supernatant was collected and quantified using an Osteocalcin SimpleStep ELISA^®^ Kit (Abcam, Cambridge, MA, USA). The amelogenin-treated group showed a higher amount of osteocalcin than the control group (n = 6, Welch’s *t*-test; * *p* < 0.05).

## 4. Discussion

Bone defects exceeding 2.0 cm or involving more than 50% of the periosteum typically do not heal spontaneously [27]. Although autologous bone grafting remains the gold standard for treating critical-sized bone defects because of its osteoconductive, osteoinductive, and osteogenic properties [28], it carries unavoidable risks such as invasiveness, pain, and infection at the donor site. Therefore, alternative therapies are urgently needed. MSCs are a promising cell source for tissue healing. Sites from which MSCs can be harvested include the bone marrow, adipose tissue, umbilical cord, and skin. However, obtaining these cells involves the harvesting of healthy tissue, which is a highly invasive process.

SHED have recently attracted attention because they can overcome the risk of invasion associated with the collection of MSCs. Given that SHED are more immature than other MSCs, they have been found to have high proliferative capacity [12], which may be advantageous for tissue regeneration. A high harvesting success rate has also been reported, thus making SHED an excellent cell source for tissue regeneration. Cell transplantation has been investigated as a potential alternative treatment to autologous bone grafting [29,30], but its inferiority in terms of residual bone mass compared with other bone substitutes suggests that stimulation with growth factors should be considered. MSCs have been investigated as an alternative therapy to cleft jaw bone grafting in a few cases, thus leaving much room for further investigation [3]. Cell proliferation and differentiation potential are also affected by the number of generations, with these properties being conserved up to the 10th generation [31,32]. In the current study, cells from passages 4–6 were used to reduce the influence of the number of passages on the osteogenic differentiation potential of SHED. Indeed, the differentiation potential of the cells appeared unaffected, as experiments on cell proliferative capacity and survival showed no significant differences in cell properties between these passages.

Humans have two non-complementary amelogenin genes, *AMELX* (Xp22.3) on the X chromosome and *AMELY* (Yp11.2) on the Y chromosome, with approximately 90% of amelogenin expression arising from *AMELX* [33]. In cleft lip and palate, a mutation in *AMELX* has been proposed as one of the contributing factors [34]. In the current study, amelogenin was investigated as a potential auxiliary factor, with consideration of its future clinical application.

We investigated the use of human full-length amelogenin as a differentiation and growth factor to stimulate SHED, recognizing that BMP2 is the leading growth factor in bone tissue engineering. The BMP family mobilizes MSCs for wound healing and osteoblast differentiation. Although the mechanism is not yet understood, BMP2 is recognized as the most effective and efficient inducer of bone differentiation [35,36,37]. The concentration of BMP2 needed to promote bone formation is known to be species-dependent and has been reported to increase in higher animals such as humans. However, there are also reports that high concentrations of BMP2 are associated with adverse effects, such as bone cysts and lipogenesis [38]. Another promising candidate growth factor is vascular endothelial growth factor (VEGF), which is expressed in various vascular tissues and is thought to promote bone regeneration by inducing angiogenesis. However, high concentrations of VEGF are associated with various adverse effects, such as vascular malformations and dysfunctional vasculature [39]. Additionally, various obstacles exist regarding its use in humans. Notably, its short half-life of 4–24 h poses a challenge because effective therapeutic dosing for bone defects is difficult to achieve without exceeding toxic concentration thresholds [40,41]. Thus, there is still room for consideration of less harmful growth factors that enhance bone regeneration in bone tissue engineering.

In this study, increased gene expression of *RUNX2*, *BGLAP*, *COL1*, and *BMP2* was observed in the amelogenin-treated group, consistent with previous reports using other MSCs [42,43]. Additionally, elevated gene expression of *CBFB*, *BMP4*, *NOTCH1*, *NOTCH2*, and *NES* was observed. *RUNX2* is a genetic marker involved in the early stages of osteoblast differentiation [44]. Additionally, CBFB protein exhibits low DNA-binding capacity independently; however, when it forms heterodimers with RUNX2 protein, its DNA-binding capacity is enhanced, playing a critical role in osteogenesis [44]. CBFB protein is required for skeletal development by regulating chondrocyte differentiation and proliferation and osteoblast differentiation. Additionally, it plays an important role in the stabilization of RUNX family proteins [45]. The gene of osteocalcin, namely *BGLAP*, was enhanced from day 18 onwards after the induction of differentiation. Osteocalcin is a marker of mature osteoblasts and is regulated by *RUNX2* [46]; our study also suggests that increased gene expression of osteocalcin may result from the upregulation of *RUNX2*.

In this study, increased gene expression of *NOTCH1*/*NOTCH2* was observed. *NOTCH1*/*NOTCH2* encode receptors of the notch signaling pathway, an evolutionarily highly conserved signaling pathway that plays a central role in cell survival, proliferation, differentiation, fate determination, and homeostasis [47]. The notch–Delta pathway has been suggested to play an important role in the proliferation and differentiation of human dental pulp stem cells [48,49]. The notch signaling pathway is activated through interactions between notch receptors (notch 1–4) and their ligands (Delta-like 1, 3, and 4 and Jagged 1 and 2). Treatment of human MSCs with Jag-1 induces osteoblastogenesis, highlighting the osteo-inductive potential of notch signaling [50]. In our study, we observed an increase in *NOTCH1*/*NOTCH2* gene expression, suggesting that the addition of amelogenin enhances the notch signaling pathway and osteoblast differentiation.

NOTCH2 protein has been reported to be absent in healthy human teeth [51]. However, following pulp tissue damage, notch signaling is activated in mesenchymal cells within the coronal portion of the pulp. This activation facilitates the differentiation of progenitor cells into odontoblast-like cells, which subsequently transition into fully differentiated odontoblasts over time, playing a protective mechanism against injury [52]. This mechanism aids in forming reparative dentin when the pulp is exposed to caries or trauma [53]. NOTCH2 protein has been identified in odontoblasts and sub-odontoblasts during dentin repair [51]. *NES*, Nestin gene marker, has been reported to be useful as a marker for odontoblasts [54], and in this study, gene expression of *NOTCH2* and *NES* increased with amelogenin treatment, suggesting the possibility of clinical application not only in bone regeneration but also in reparative dentin formation using the pulp capping method. Indeed, amelogenin capping has been reported to have a favorable healing course in vivo [55,56].

The observed changes in gene expression suggest that amelogenin treatment promotes osteoblast differentiation, highlighting its strong potential for SHED application in tissue engineering. By activating specific genes that support bone formation, amelogenin enables efficient bone regeneration. In particular, the increased expression of osteocalcin indicates that the final bone formation is progressing, and ELISA evaluation showed increased protein levels.

ALP activity, Alizarin Red S staining, and calcium quantification also revealed a significant increase in the amelogenin-treated group compared with the control group, consistent with previous reports using other MSCs [18]. Increased calcification and calcium content by ALP activity and Alizarin Red S staining indicated enhanced bone formation, supporting the possibility that amelogenin in combination with SHED may enhance the efficiency of bone tissue regeneration. These results suggest that amelogenin may be a useful adjunct in SHED-based bone tissue engineering.

This in vitro study had a few limitations. Although this study used SHED, caution should be exercised when interpreting the results in comparison with other cell sources. Comparative studies with other stem cells are required to determine how the specific properties of SHED may affect their ability to form bones. It has been reported that MSCs are heterogeneous and form heterogenous cell populations. Moreover, MSCs isolated from different donors and cell sources have different biological properties [57]. Even within MSC populations that share similarities, MSCs can be classified into different subpopulations according to the expression of cell surface markers and their functions [58]. Therefore, the results of the experiments obtained should be considered with regard to the type of MSC and their cell populations. Several mechanisms of action for amelogenin have been reported; however, no pathways were identified in this study. Further studies should focus on clarifying how amelogenin affects signaling pathways and how it interacts with other factors. The present study was limited to in vitro experiments, and animal and human clinical trials are required to assess its efficacy and safety in clinical practice. Although this study achieved results using short-term cell culture, the long-term effects and safety of amelogenin in tissue regeneration and bone repair were not assessed. Long-term follow-up studies are necessary to support the clinical application of regenerative medicine. Future studies should also focus on understanding the effects of SHED on surface antigens and their expression across individual cell populations. Additionally, further investigations into the mechanism of action of amelogenin and its combination with SHED in models of bone loss are essential to identify optimal treatment strategies and advance clinical trials of bone regeneration therapies.

## 5. Conclusions

The combination of SHED and amelogenin may be effective for bone regeneration, suggesting the possibility of a new approach in regenerative medicine. In future studies, bone regeneration therapy using SHED and amelogenin is expected to become an effective treatment for refractory bone defects, such as CLP and jawbone reconstruction.

## Figures and Tables

**Table 1:** Primer sequences used in this study.

Gene		Sequence (5′ → 3′)
*GAPDH*	Forward	ATG GCC TTC CGT GTT CCT
	Reverse	CCC AAG ATG CCC TTC AGT
*RUNX2*	Forward	CAC TGG CGC TGC AAC AAG A
	Reverse	CAT TCC GGA GCT CAG CAG AAT AA
*CBFB*	Forward	AGA AGC AAG TTC GAG AAC GAG
	Reverse	CCT GAA GCC CGT GTA CTT AAT CT
*COL1*	Forward	CCC GGG TTT CAG AGA CAA CTT C
	Reverse	TCC ACA TGC TTT ATT CCA GCA ATC
*BMP2*	Forward	CTG GCT GAT CAT CTG AAC TCC ACT A
	Reverse	TCG GGA CAC AGC ATG CCT TA
*BMP4*	Forward	AGA TCC ACA GCA CTG GTC TTG AGT A
	Reverse	TCT CAG GGA TGC TGC TGA GG
*BGLAP*	Forward	GAC TGT GAC GAG TTG GCT GA
	Reverse	GAA GAG GAA AGA AGG GTG CC
*NOTCH1*	Forward	GAG GCG TGG CAG ACT ATG C
	Reverse	CTT GTA CTC CGT CAG CGT GA
*NOTCH2*	Forward	CAA CCG CAA TGG AGG CTA TG
	Reverse	GCG AAG GCA CAA TCA TCA ATG TT
*NES*	Forward	CAA CAG CGA CGG AGG TCT C
	Reverse	GCC TCT ACG CTC TCT TCT TTG A

## Data Availability

The data that support the findings of this study are available from the corresponding author upon reasonable request.

## Supplementary Materials

The following supporting information can be downloaded at: https://www.mdpi.com/article/10.3390/cells14090657/s1, File S1: Alizarin red S staining. Cacification Evaluation (Alizarin red S staining absorbance). ALP staining.

## Abbreviations

The following abbreviations are used in this manuscript:

CLP	Cleft palate
MSCs	Mesenchymal stem cells
SHED	Stem cells from human exfoliated deciduous teeth
EMD	Enamel matrix derivative
PBS	Phosphate-buffered saline
α-MEM	Minimal essential medium eagle
FBS	Fetal bovine serum
ALP	Alkaline phosphatase
VEGF	Vascular endothelial growth factor

## References

[1] Schroeder J.E., Mosheiff R. (2011). Tissue engineering approaches for bone repair: Concepts and evidence. Injury.

[2] Murthy A.S., Lehman J.A. (2005). Evaluation of alveolar bone grafting: A survey of ACPA teams. Cleft Palate Craniofac. J..

[3] Dissaux C., Ruffenach L., Bruant-Rodier C., George D., Bodin F., Rémond Y. (2022). Cleft alveolar bone graft materials: Literature review. Cleft Palate Craniofac. J..

[4] Tournier P., Guicheux J., Paré A., Veziers J., Barbeito A., Bardonnet R., Corre P., Geoffroy V., Weiss P., Gaudin A. (2021). An extrudable partially demineralized allogeneic bone paste exhibits a similar bone healing capacity as the “gold standard” bone graft. Front. Bioeng. Biotechnol..

[5] Raposo-Amaral C.A., Denadai R., Chammas D.Z., Marques F.F., Pinho A.S., Roberto W.M., Buzzo C.L., Raposo-Amaral C.E. (2015). Cleft patient-reported postoperative donor site pain following alveolar autologous iliac crest bone grafting: Comparing two minimally invasive harvesting techniques. J. Craniofac. Surg..

[6] Faour O., Dimitriou R., Cousins C.A., Giannoudis P.V. (2011). The use of bone graft substitutes in large cancellous voids: Any specific needs?. Injury.

[7] Oliveira É.R., Nie L., Podstawczyk D., Allahbakhsh A., Ratnayake J., Brasil D.L., Shavandi A. (2021). Advances in growth factor delivery for bone tissue engineering. Int. J. Mol. Sci..

[8] Bose S., Roy M., Bandyopadhyay A. (2012). Recent advances in bone tissue engineering scaffolds. Trends Biotechnol..

[9] Friedenstein A.J., Chailakhjan R.K., Lalykina K.S. (1970). The development of fibroblast colonies in monolayer cultures of guinea-pig bone marrow and spleen cells. Cell Tissue Kinet..

[10] Seong J.M., Kim B.C., Park J.H., Kwon I.K., Mantalaris A., Hwang Y.S. (2010). Stem cells in bone tissue engineering. Biomed. Mater..

[11] Zha K., Tian Y., Panayi A.C., Mi B., Liu G. (2022). Recent advances in enhancement strategies for osteogenic differentiation of mesenchymal stem cells in bone tissue engineering. Front. Cell Dev. Biol..

[12] Miura M., Gronthos S., Zhao M., Lu B., Fisher L.W., Robey P.G., Shi S. (2003). SHED: Stem cells from human exfoliated deciduous teeth. Proc. Natl. Acad. Sci. USA.

[13] Rajendran R., Gopal S., Masood H., Vivek P., Deb K. (2013). Regenerative potential of dental pulp mesenchymal stem cells harvested from high caries patient’s teeth. J. Stem Cells.

[14] Nakajima K., Kunimatsu R., Ando K., Hiraki T., Rikitake K., Tsuka Y., Abe T., Tanimoto K. (2019). Success rates in isolating mesenchymal stem cells from permanent and deciduous teeth. Sci. Rep..

[15] Cheng L., Li Y., Xia Q., Meng M., Ye Z., Tang Z., Feng H., Chen X., Chen H., Zeng X. (2021). Enamel matrix derivative (EMD) enhances the osteogenic differentiation of bone marrow mesenchymal stem cells (BMSCs). Bioengineered.

[16] Mrozik K.M., Gronthos S., Menicanin D., Marino V., Bartold P.M. (2012). Effect of coating Straumann Bone Ceramic with Emdogain on mesenchymal stromal cell hard tissue formation. Clin. Oral. Investig..

[17] Sculean A., Windisch P., Döri F., Keglevich T., Molnár B., Gera I. (2007). Emdogain in regenerative periodontal therapy. A review of the literature. Fogorv. Sz..

[18] Tanimoto K., Huang Y.C., Tanne Y., Kunimatsu R., Michida M., Yoshioka M., Ozaki N., Sasamoto T., Yoshimi Y., Kato Y. (2012). Amelogenin enhances the osteogenic differentiation of mesenchymal stem cells derived from bone marrow. Cells Tissues Organs.

[19] Olivares-Navarrete R., Vesper K., Hyzy S.L., Almaguer-Flores A., Boyan B.D., Schwartz Z. (2014). Role of the N-terminal peptide of amelogenin on osteoblastic differentiation of human mesenchymal stem cells. Eur. Cells Mater..

[20] Kunimatsu R., Awada T., Yoshimi Y., Ando K., Hirose N., Tanne Y., Sumi K., Tanimoto K. (2018). The C-terminus of the amelogenin peptide influences the proliferation of human bone marrow mesenchymal stem cells. J. Periodontol..

[21] Huang Y.C., Tanimoto K., Tanne Y., Kamiya T., Kunimatsu R., Michida M., Yoshioka M., Yoshimi Y., Kato Y., Tanne K. (2010). Effects of human full-length amelogenin on the proliferation of human mesenchymal stem cells derived from bone marrow. Cell Tissue Res..

[22] Carneiro K.M.M., Zhai H., Zhu L., Horst J.A., Sitlin M., Nguyen M., Wagner M., Simpliciano C., Milder M., Chen C.-L. (2016). Amyloid-like ribbons of amelogenins in enamel mineralization. Sci. Rep..

[23] Vowden P., Romanelli M., Peter R., Boström A., Josefsson A., Stege H. (2006). The effect of amelogenins (Xelma) on hard-to-heal venous leg ulcers. Wound Repair. Regen..

[24] Gronthos S., Mankani M., Brahim J., Robey P.G., Shi S. (2000). Postnatal human dental pulp stem cells (DPSCs) in vitro and in vivo. Proc. Natl. Acad. Sci. USA.

[25] Nakajima K., Kunimatsu R., Ando K., Hiraki T., Abe T., Tsuka Y., Hiraki A., Tanimoto K. (2018). Comparison of the bone regeneration ability between stem cells from human exfoliated deciduous teeth, human dental pulp stem cells and human bone marrow mesenchymal stem cells. Biochem. Biophys. Res. Commun..

[26] Kunimatsu R., Rikitake K., Yoshimi Y., Putranti N.A.R., Hayashi Y., Tanimoto K. (2023). Bone Differentiation Ability of CD146-Positive Stem Cells from Human Exfoliated Deciduous Teeth. Int. J. Mol. Sci..

[27] Keating J.F., Simpson A.H.R.W., Robinson C.M. (2005). The management of fractures with bone loss. J. Bone Jt. Surg. Br..

[28] Sen M.K., Miclau T. (2007). Autologous iliac crest bone graft: Should it still be the gold standard for treating nonunions?. Injury.

[29] Al-Ahmady H.H., Abd Elazeem A.F., Bellah Ahmed N.E.M., Shawkat W.M., Elmasry M., Abdelrahman M.A., Abderazik M.A. (2018). Combining autologous bone marrow mononuclear cells seeded on collagen sponge with nano hydroxyapatite, and platelet-rich fibrin: Reporting a novel strategy for alveolar cleft bone regeneration. J. Craniomaxillofac. Surg..

[30] Pradel W., Lauer G. (2012). Tissue-engineered bone grafts for osteoplasty in patients with cleft alveolus. Ann. Anat..

[31] Neuhuber B., Swanger S.A., Howard L., Mackay A., Fischer I. (2008). Effects of plating density and culture time on bone marrow stromal cell characteristics. Exp. Hematol..

[32] Briquet A., Dubois S., Bekaert S., Dolhet M., Beguin Y., Gothot A. (2010). Prolonged ex vivo culture of human bone marrow mesenchymal stem cells influences their supportive activity toward NOD/SCID-repopulating cells and committed progenitor cells of B lymphoid and myeloid lineages. Haematologica.

[33] Hu J.C.C., Chan H.C., Simmer S.G., Seymen F., Richardson A.S., Hu Y., Milkovich R.N., Estrella N.M., Yildirim M., Bayram M. (2012). Amelogenesis imperfecta in two families with defined AMELX deletions in ARHGAP6. PLoS ONE.

[34] Oliveira F.V., Dionísio T.J., Neves L.T., Machado M.A.A.M., Santos C.F., Oliveira T.M. (2014). Amelogenin gene influence on enamel defects of cleft lip and palate patients. Braz. Oral Res..

[35] Salgado A.J., Coutinho O.P., Reis R.L. (2004). Bone tissue engineering: State of the art and future trends. Macromol. Biosci..

[36] Bai Y., Li P., Yin G., Huang Z., Liao X., Chen X., Yao Y. (2013). BMP-2, VEGF and bFGF synergistically promote the osteogenic differentiation of rat bone marrow-derived mesenchymal stem cells. Biotechnol. Lett..

[37] Wang W., Yeung K.W.K. (2017). Bone grafts and biomaterials substitutes for bone defect repair: A review. Bioact. Mater..

[38] Bao X., Zhu L., Huang X., Tang D., He D., Shi J., Xu G. (2017). 3D biomimetic artificial bone scaffolds with dual-cytokines spatiotemporal delivery for large weight-bearing bone defect repair. Sci. Rep..

[39] Dreyer C.H., Kjaergaard K., Ding M., Qin L. (2020). Vascular endothelial growth factor for in vivo bone formation: A systematic review. J. Orthop. Transl..

[40] Jin Y., Guo Y.H., Li J.C., Li Q., Ye D., Zhang X.X., Li J.T. (2023). Vascular endothelial growth factor protein and gene delivery by novel nanomaterials for promoting liver regeneration after partial hepatectomy. World J. Gastroenterol..

[41] Eckardt H., Ding M., Lind M., Hansen E.S., Christensen K.S., Hvid I. (2005). Recombinant human vascular endothelial growth factor enhances bone healing in an experimental nonunion model. J. Bone Jt. Surg. Br..

[42] Hu J., Rong S., Zhongchen S., Lan C. (2011). Human amelogenin up-regulates osteogenic gene expression in human bone marrow stroma cells. Biochem. Biophys. Res. Commun..

[43] Hsia T.L., Lin Z., Xia Y., Shu R., Xie Y. (2024). A photoresponsive recombinant human amelogenin-loaded hyaluronic acid hydrogel promotes bone regeneration. J. Periodontal Res..

[44] Komori T. (2020). Molecular mechanism of Runx2-dependent bone development. Mol. Cells.

[45] Komori T. (2003). Requisite roles of Runx2 and Cbfb in skeletal development. J. Bone Miner. Metab..

[46] Komori T. (2019). Regulation of proliferation, differentiation and functions of osteoblasts by Runx2. Int. J. Mol. Sci..

[47] Majidinia M., Alizadeh E., Yousefi B., Akbarzadeh M., Mihanfar A., Rahmati-Yamchi M., Zarghami N. (2017). Co-inhibition of Notch and NF-κB signaling pathway decreases proliferation through downregulating IκB-α and Hes-1 expression in human ovarian cancer OVCAR-3 cells. Drug Res..

[48] Wang X.F., Zhang G., Qiu S.B., He F., Tan Y.H., Chen Q. (2011). Effect of Notch ligand Delta1-RNA interference by lentivirus on proliferation and differentiation of human dental pulp stem cells. Zhonghua Kou Qiang Yi Xue Za Zhi.

[49] Sawangmake C., Nowwarote N., Pavasant P., Chansiripornchai P., Osathanon T. (2014). A feasibility study of an in vitro differentiation potential toward insulin-producing cells by dental tissue-derived mesenchymal stem cells. Biochem. Biophys. Res. Commun..

[50] Zhu F., Sweetwyne M.T., Hankenson K.D. (2013). PKCδ is required for Jagged-1 induction of human mesenchymal stem cell osteogenic differentiation. Stem Cells.

[51] Mitsiadis T.A., Roméas A., Lendahl U., Sharpe P.T., Farges J.C. (2003). Notch2 protein distribution in human teeth under normal and pathological conditions. Exp. Cell Res..

[52] Ma L., Wang S.C., Tong J., Hu Y., Zhang Y.Q., Yu Q. (2016). Activation and dynamic expression of Notch signalling in dental pulp cells after injury in vitro and in vivo. Int. Endod. J..

[53] Tziafas D., Smith A.J., Lesot H. (2000). Designing new treatment strategies in vital pulp therapy. J. Dent..

[54] Quispe-Salcedo A., Ida-Yonemochi H., Nakatomi M., Ohshima H. (2012). Expression patterns of nestin and dentin sialoprotein during dentinogenesis in mice. Biomed. Res..

[55] Bahammam L.A., Alsharqawi W., Bahammam H.A., Mounir M. (2024). Histological evaluation of pulpal response and dentin bridge formation after direct pulp capping using recombinant amelogenin and mineral trioxide aggregate (MTA). Curēus.

[56] Peng X., Han S., Wang K., Ding L., Liu Z., Zhang L. (2021). Evaluating the potential of an amelogenin-derived peptide in tertiary dentin formation. Regen. Biomater..

[57] Wang W., Han Z.C. (2019). Heterogeneity of human mesenchymal stromal/stem cells. Adv. Exp. Med. Biol..

[58] Johnson K.W., Dooner M., Quesenberry P.J. (2007). Fluorescence activated cell sorting: A window on the stem cell. Curr. Pharm. Biotechnol..

