# Supplementary material for: Effect of Recombinant Human Amelogenin on the Osteogenic Differentiation Potential of SHED
Source: Cells. 2025 Apr 30;14(9):657. doi: 10.3390/cells14090657 (PMC12071429; doi:10.3390/cells14090657)

#【Supplemental data】

Alizarin red S staining

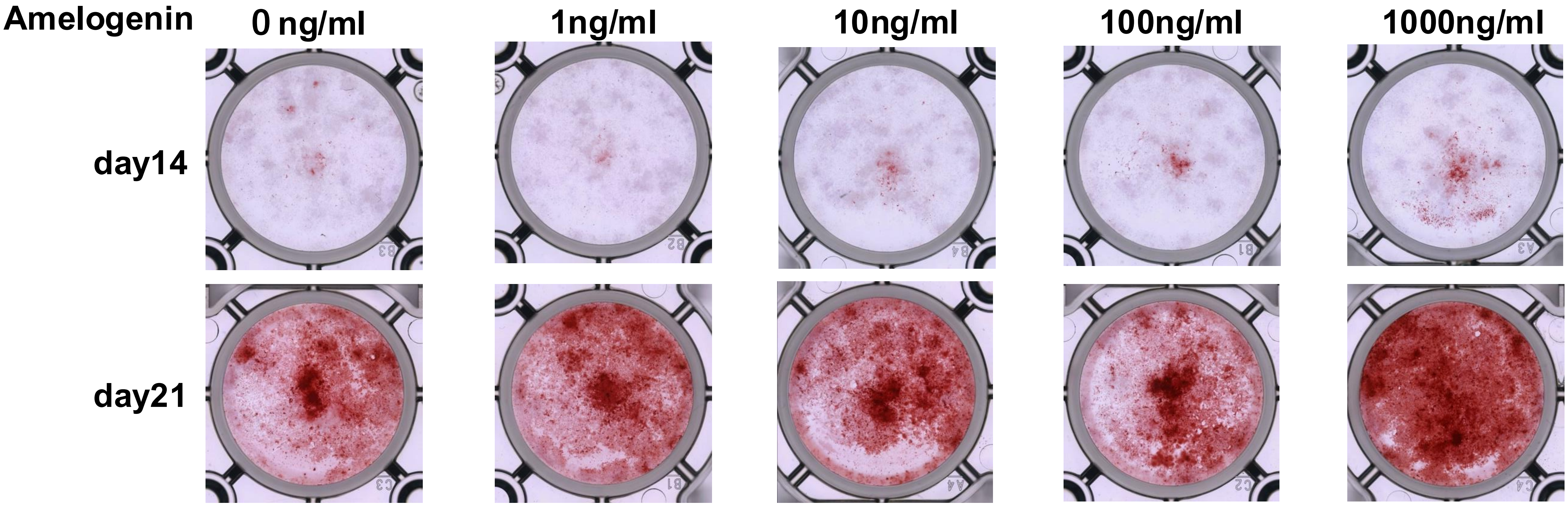

#【Supplemental data】

Alizarin red S staining

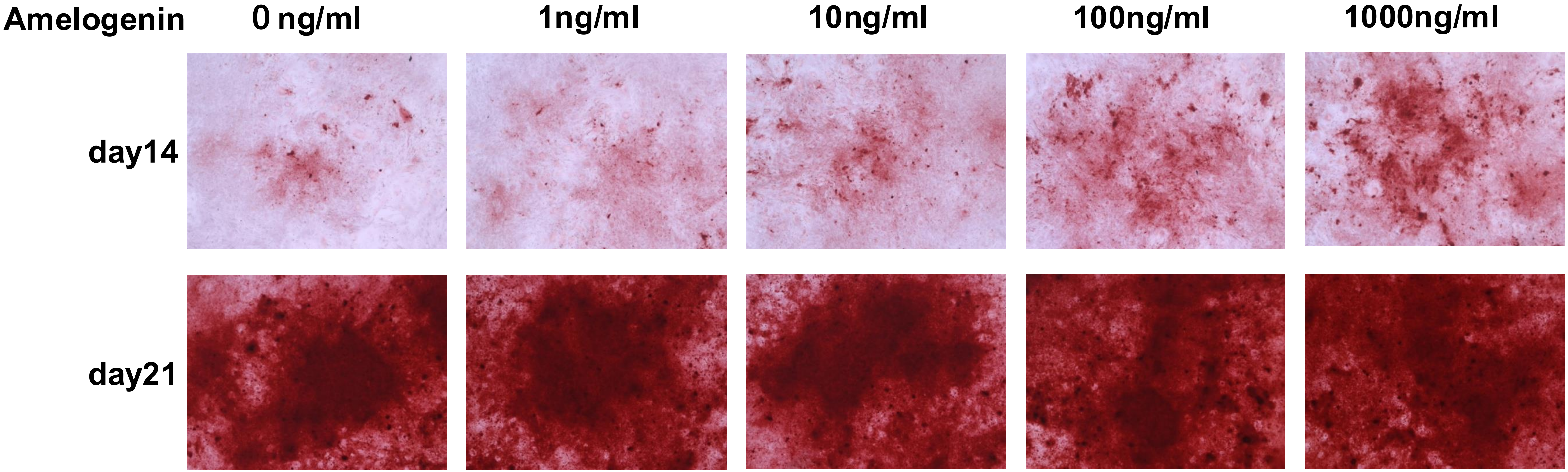

【Supplemental data】

Cacification Evaluation (Alizarin red S staining absorbance)

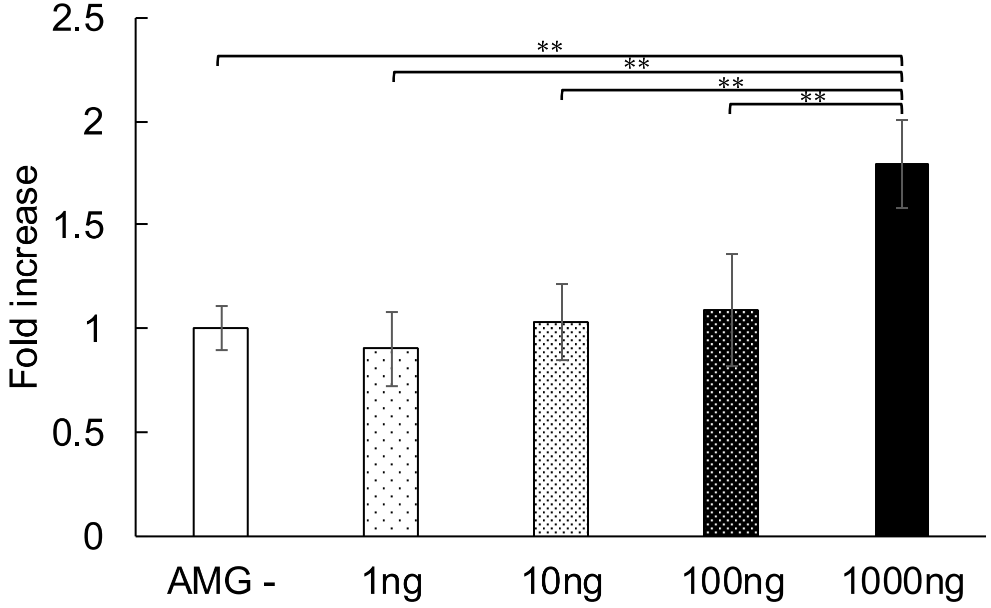

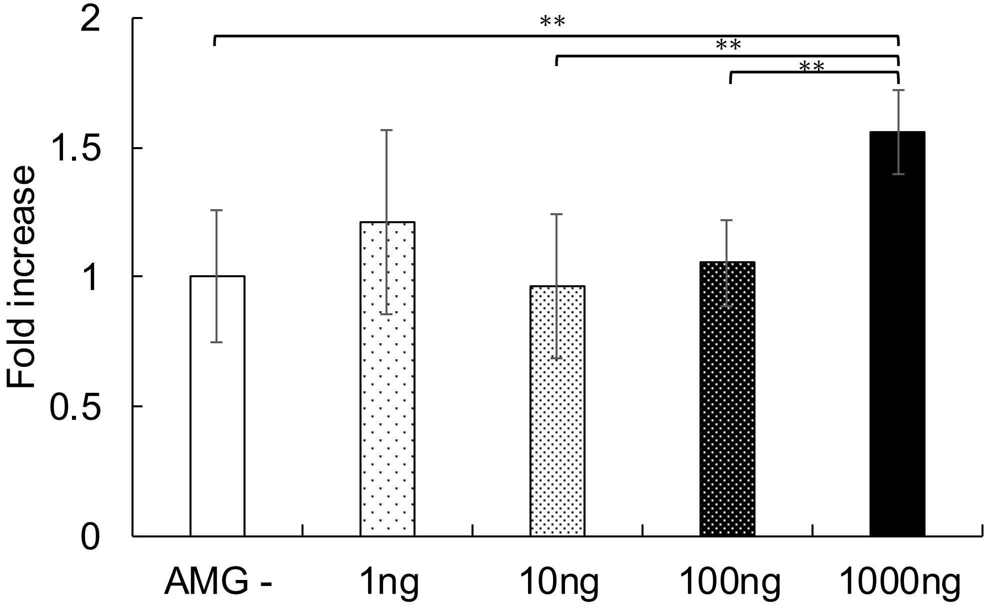

n = 6,Tukey’s HSD test, **p<0.01

【Supplemental data】

ALP staining

Amelogenin **0 ng/ml** **1ng/ml** **10ng/ml** **100ng/ml** **1000ng/ml**

day7

day14

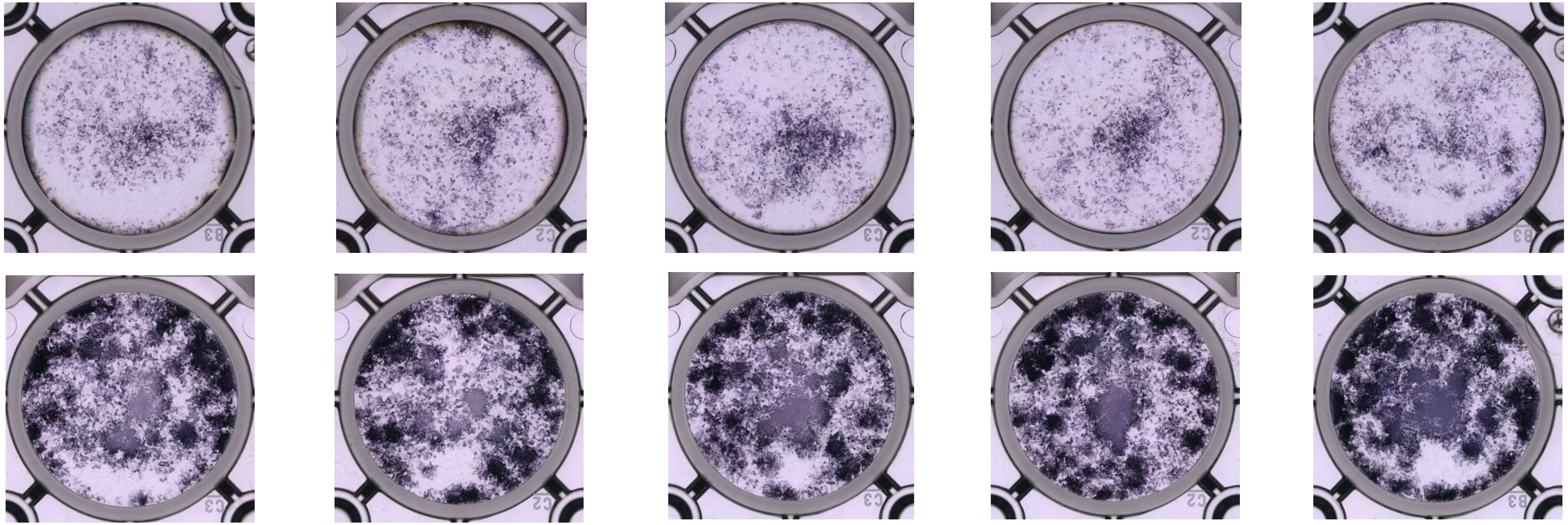

#【Supplemental data】

ALP staining

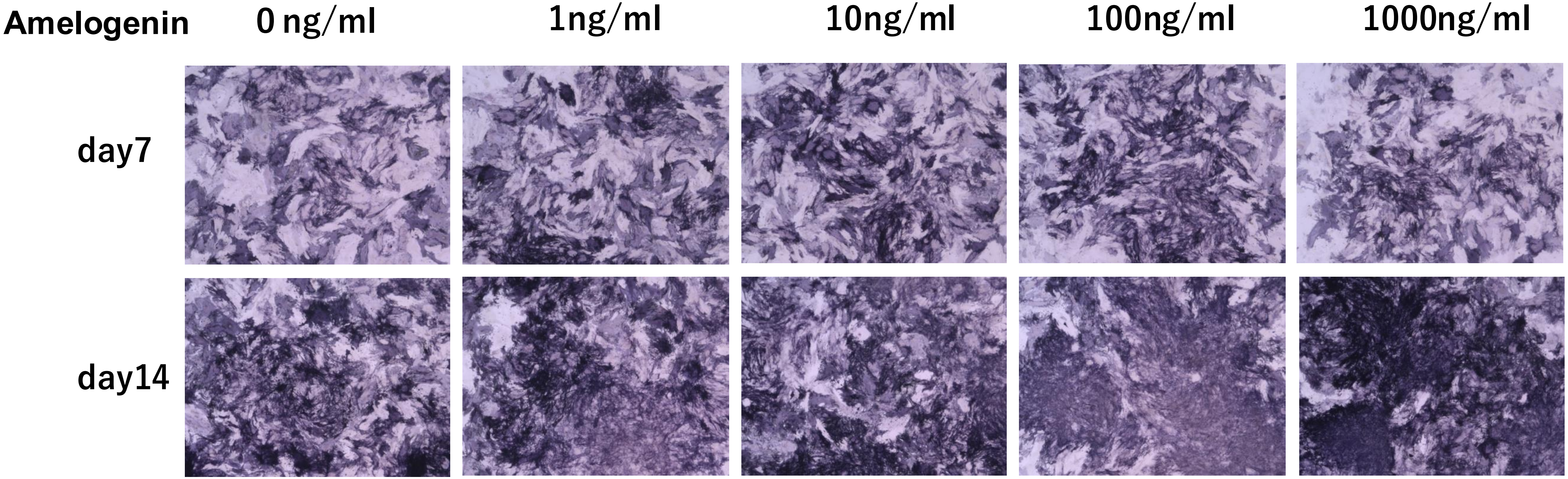

Supplement: Supplementary file 1 [file cells-14-00657-s001.zip › cells-3583426-supplementary.pdf]
